# Visceral Artery Injuries during Nephrectomy: A Systematic Review of Published Cases

**DOI:** 10.1016/j.ejvsvf.2026.07.004

**Published:** 2026-07-12

**Authors:** Marion Cano, Anne Lejay, Hervé Lang, Nabil Chakfé

**Affiliations:** a Department of Vascular Surgery, Kidney Transplantation and Innovation, Strasbourg University Hospital, Strasbourg, France; b Department of Urology, Strasbourg University Hospital, Strasbourg, France

**Keywords:** Coeliac artery, Iatrogenic disease, Intra-operative complications, Superior mesenteric artery

## Abstract

**Introduction:**

Visceral artery injuries during nephrectomy, such as superior mesenteric artery (SMA) and coeliac artery (CA) injuries, are rare but potentially lethal complications. This systematic review was performed with a focus on visceral artery injuries during nephrectomy in order to describe how to prevent and manage SMA or CA injuries.

**Method:**

A systematic literature review was performed in Medline and the Cochrane Library from inception to December 2025 to identify published case reports and case series of SMA or CA injuries during nephrectomy. MeSH terms used were: “mesenteric artery, superior” “coeliac artery”, “intra-operative complications”, and “iatrogenic disease”. Outcomes of interest were initial operative indication, cause and type of injury, anatomic risk factors, timing of diagnosis, type of vascular repair, and patient outcome. Quality of the studies was assessed according to the CARE guidelines.

**Results:**

After duplicate removal, screening, and full text eligibility assessment, 16 publications of varying quality reporting 20 cases of SMA and or CA injury during nephrectomy were included. All injuries occurred during left nephrectomy. Injury involved the SMA in 16 cases (80%) and both the SMA and CA in four cases (20%). The dominant mechanism was anatomic misperception: the SMA or CA was mistaken for the left renal artery, facilitated by bulky lymphadenopathy, large tumour size (median 12 cm), and post-inflammatory adhesions. SMA and CA injury was recognised intra-operatively in 16 cases (80%), while delayed diagnosis (ranging 12 – 48 hours) occurred in four cases (20%) and was associated with worse outcomes. Revascularisation was performed in 18 cases (90%), mostly by primary end to end anastomosis of the injured artery. Three patients died (15%).

**Conclusion:**

Visceral artery injuries during nephrectomy carry a substantial mortality risk, especially when the diagnosis is delayed. Careful pre-operative computed tomography angiography analysis, prompt injury identification, and immediate vascular surgical support are central elements of prevention and management.

## Highlights

• Visceral artery injuries during nephrectomy are rare but devastating.
• Prompt recognition is mandatory.
• Prompt vascular surgeon assistance is required.
• Vascular repair depends on the type of injury.
• Autologous material is preferred for reconstruction.

## Introduction

Nephrectomy, whether total or partial, is among the most commonly performed urological procedures. Nephrectomy can be performed for various reasons, including renal tumours, chronic kidney disease, severe kidney injury, or for live donor. Injury to the superior mesenteric artery (SMA) or coeliac artery (CA) during nephrectomy is a rare but potentially devastating complication.^1^ Only a few cases have been described, but injuries to the CA or SMA might be under reported.^2^

SMA or CA injury during nephrectomy most commonly occurs with inadvertent injury to or mistaken ligation of the SMA or CA instead of the renal artery.^3^^,^^4^ SMA or CA injury can lead to significant morbidity and mortality, affecting patient outcomes, since the SMA and CA play vital roles in supplying blood to the stomach, duodenum, small bowel, proximal large bowel, liver, pancreas, and spleen. Failure to recognise and repair injured vessels results in ischaemia, organ dysfunction, and death for most patients. Therefore, a comprehensive understanding of the anatomy, mechanisms of injury, clinical implications, and repair strategies is essential so that surgeons can recognise and manage this potentially life threatening complication.

This review aimed to identify all published case reports and case series of SMA and or CA injury during nephrectomy, with a focus on the anatomic and operative conditions predisposing to vessels injuries, but also clinical presentation, time to diagnosis, type of repair, and patient outcome. By synthesising this information, the authors sought to describe how to prevent and also how to manage SMA and or CA injury in a standardised stepwise manner.

## Study design

A systematic literature search was conducted in Medline and the Cochrane Library, from inception to December 2025, to identify published case reports and case series of SMA and or CA injury during nephrectomy.

## Method

### Search strategy

A combined search strategy of MeSH terms “mesenteric artery, superior” “coeliac artery”, “intra-operative complications”, and “iatrogenic disease” was used. Two investigators (M.C., A.L.) screened all titles and abstracts collected from the search strategy for relevance. The first 20 related items of all relevant articles were scanned for other potentially relevant studies. The full texts of all relevant articles were obtained and reviewed for suitability independently by both reviewers. The reference lists of each article were scanned for other potentially relevant studies. Any disagreements about study inclusion was resolved by consensus.

### Inclusion and exclusion criteria

The included studies were English full text. All studies reporting SMA or CA injury occurring during nephrectomy, regardless of surgical approach, were included. Experimental studies, anatomic studies, or studies reporting isolated renal artery injuries were excluded.

### Study records

The same reviewers independently extracted data using a standardised form. This was performed in duplicate to increase accuracy and reduce measurement bias. Any disagreement in data collection was resolved by consensus.

### Data items

Extracted data included study characteristics (year of publication, study design, number of cases), and case description with surgical approach (open, laparoscopic, or robot assisted), operative indication (oncological or benign), type of injury, risk factors, timing of diagnosis, clinical presentation, treatment, and patient outcome.

### Assessment of the methodological quality of the studies

The CARE (CAse REports) guidelines were used to assess the methodological quality of the included studies.^5^ Each included study was rated from one to 13: one point was awarded each time one of the 13 items of the CARE checklist was adequately addressed.

## Results

### Overview of included studies

**Figure 1:**
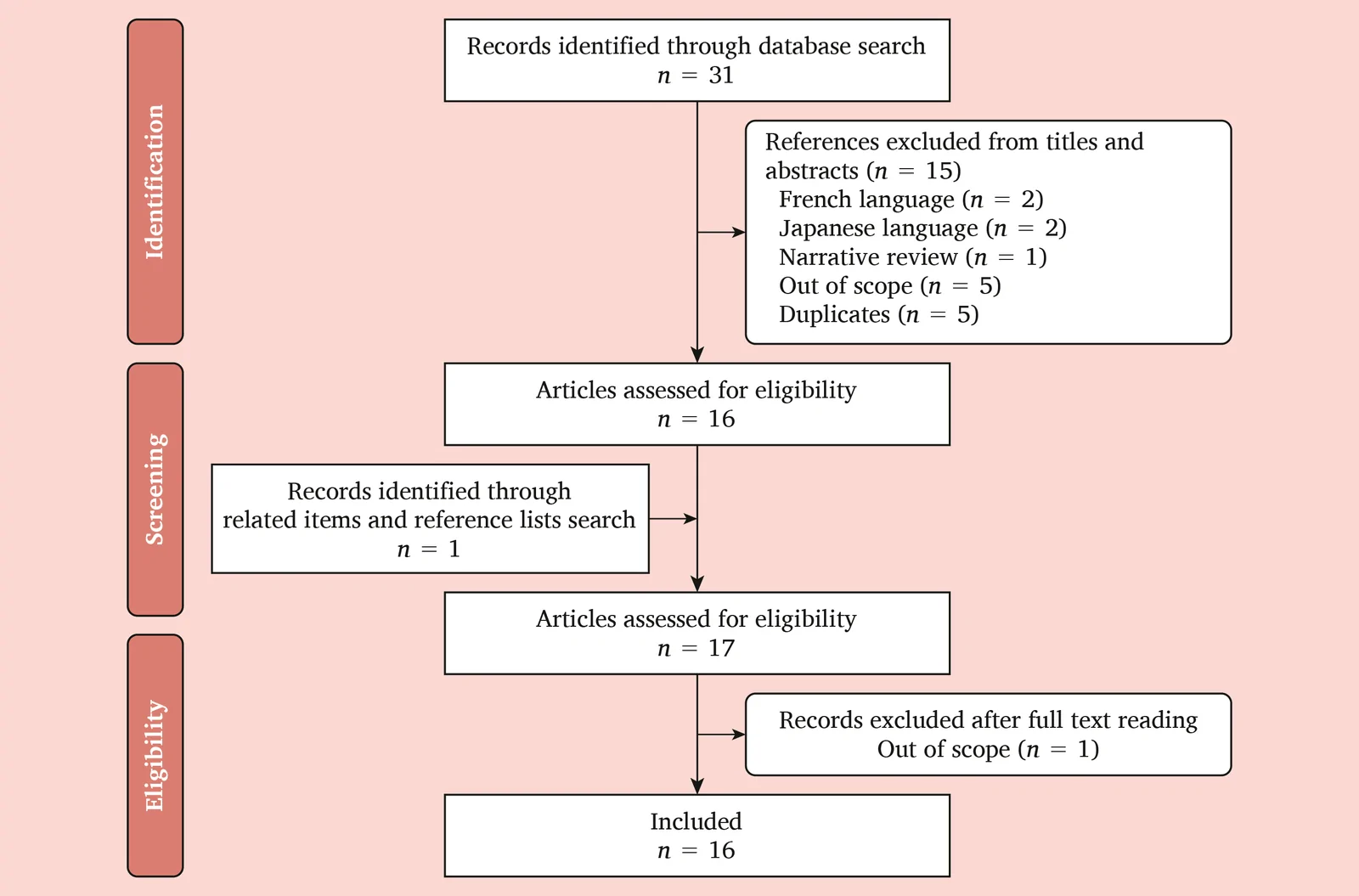
Flowchart showing study selection.

After duplicate removal, screening, and full text eligibility assessment, 16 publications were included, reporting 20 cases of SMA and or CA injury during nephrectomy.^6^, ^7^, ^8^, ^9^, ^10^, ^11^, ^12^, ^13^, ^14^, ^15^, ^16^, ^17^, ^18^, ^19^, ^20^, ^21^ A flowchart showing study selection is provided in Figure 1. Publications spanned 1992 – 2025; most were case reports (*n* = 14) or small case series (*n* = 2). The largest series reported four paediatric cases from a national registry.^6^ A summary of study characteristics, surgical approach, operative indication, type of injury and risk factors, timing of diagnosis, clinical presentation, treatment, and patient outcomes is presented in Table 1.

**Table 1:** Summary of study characteristics, surgical approaches, operative indications, types of injury and risk factors, timing of diagnosis, clinical presentations, treatment, and patient outcomes.

Author, year, CARE score	Approach	Cases – *n*	Type of injury	Timing of diagnosis	Risk factors	Clinical presentation	Treatment	Outcome
Ritchey 1992^6^ 5/13	Open Open Open Open	Four (paediatric patients)	SMA SMA SMA SMA	Intra-operative Intra-operative Intra-operative Intra-operative	Bulky tumour Bulky tumour Bulky tumour Bulky tumour	NA NA NA NA	Primary end to end anastomosis Primary end to end anastomosis Primary end to end anastomosis End to end anastomosis with internal iliac artery interposition	Alive Alive Alive Alive
Siqueira 2002^7^ 4/13	Laparoscopy	One	SMA	Intra-operative	Bulky tumour	NA	Primary end to end anastomosis	Dead
Kumar 2007^8^ 6/13	Open	One	SMA	Intra-operative	Bulky tumour	NA	Primary end to end anastomosis	Alive
Blunt 2008^9^ 6/13	Open	One	SMA	Intra-operative	Xanthogranulomatous pyelonephritis	NA	Venous patch repair (left renal vein)	Alive
Nevoux 2008^10^ 7/13	Open Laparoscopy	One One	SMA + CA SMA + CA	Intra-operative 48 hours	Bulky tumour Adhesions to surrounding tissues	NA Abdominal pain and acidosis	Primary end to end anastomosis Primary end to end anastomosis	Alive Dead
Abu-Ghazala 2010^11^ 8/13	Laparoscopy	One	SMA + CA	Intra-operative	Bulky tumour	NA	CA bypass with the splenic artery, SMA bypass with the saphenous vein	Alive
Afonso 2019^12^ 7/13	Open	One	SMA	Intra-operative	Bulky tumour	NA	SMA bypass with the splenic artery	Alive
Kumar 2019^13^ 7/13	Robotic	One	SMA	Intra-operative	Adhesions to surrounding tissues	NA	Primary end to end anastomosis	Alive
Zhang 2020^14^ 6/13	Open	One	SMA	Intra-operative	Bulky tumour	NA	End to end anastomosis using the renal artery stump	Alive
Deb 2021^15^ 7/13	Open	One (paediatric patient)	SMA	12 hours	Bulky tumour	Acidosis	Primary end to end anastomosis	Alive
Mayor 2022^16^ 6/13	Robotic	One	SMA	Intra-operative	Bulky tumour	NA	Primary end to end anastomosis	Alive
Ashraf 2022^17^ 7/13	Open	One	SMA	24 hours	Bulky tumour	Ileus and vomiting	Conservative	Alive
Sayegh 2023^18^ 6/13	Robotic	One	SMA	Intra-operative	Bulky tumour	NA	Primary end to end anastomosis	Alive
Huangh 2024^19^ 8/13	Open	One	SMA	Intra-operative	Bulky tumour	NA	Primary end to end anastomosis	Alive
Wainwright 2024^20^ 7/13	Robotic	One	SMA	Intra-operative	Bulky tumour	NA	Primary end to end anastomosis	Alive
Anwar 2025^21^ 7/13	Open	One	SMA + CA	36 hours	Bulky tumour	Multiorgan failure	None	Death

### Surgical approach

All reported cases involved the left kidney, consistent with the anatomic proximity of the SMA, CA, and left renal artery (LRA). The surgical approach was open in 13 cases (65%), robotic in four cases (20%), and laparoscopic in three cases (15%).

### Operative indication

The operative indication was oncological in 17 cases (85%), and benign (xanthogranulomatous pyelonephritis, inflammatory renal disease) in three cases (15%). Tumour size was reported in 16 cases and ranged 7 – 19.5 cm (median 12 cm). Locally advanced disease with bulky retroperitoneal or peri-hilar lymphadenopathy was documented in nine cases (45%).

### Type of injury and risk factors

Injury involved the SMA in 16 cases (80%) and both the SMA and CA in four cases (20%). Identified anatomic risk factors included: bulky para-aortic or hilar lymphadenopathy or tumour distorting the SMA in 17 cases (85%), and post-inflammatory or post-surgical dense adhesions in three cases (15%). The mechanism of injury was mostly anatomic misperception: the SMA and or CA were mistaken for the LRA and clipped or ligated.

### Timing of diagnosis and clinical presentation

The injury was recognised intra-operatively in 16 cases (80%). Delayed diagnosis occurred in four cases (20%), with delays ranging 12 – 48 hours post-operatively. In these four cases of delayed diagnosis, the post-operative presentation was non-specific. Metabolic acidosis was attributed to post-operative renal failure, and dialysis was initiated. The diagnosis was made since progressive metabolic derangement with refractory lactic acidosis and hyperkalaemia continued over the following hours.

### Treatment

Revascularisation was attempted in 18 cases (90%), including 15 SMA injuries and three SMA and CA injuries, corresponding to a total of 21 arterial reconstructions. The type of repair varied depending on the type of injury, length of injured segment, and available possibilities for repair (Table 2).

**Table 2:** Type of repair depending on the type of injury.

Type of injury	Type of repair	Number of vascular reconstructions	Reference
SMA alone	Primary end to end anastomosis	11	Ritchey 1992^6^ Siqueira 2022^7^ Kumar 2007^8^ Kumar 2019^13^ Deb 2021^15^ Mayor 2022^16^ Sayegh 2023^18^ Huangh 2024^19^ Wainwright 2024^20^
	End to end anastomosis with internal iliac artery interposition	One	Ritchey 1992^6^
	Left renal vein patch repair	One	Blunt 2008^9^
	Bypass using the splenic artery	One	Afonso 2019^12^
	End to end anastomosis using the renal artery stump	One	Zhang 2020^14^
SMA and CA	Primary end to end anastomosis for SMA Primary end to end anastomosis for CA	Two Two	Nevoux 2008^10^
	CA bypass with the splenic artery SMA bypass with the saphenous vein	One One	Abu-Ghazala 2010^11^

Primary end to end anastomosis was performed in 15 cases, end to end anastomosis with internal iliac artery interposition in one case, end to end anastomosis using the renal artery stump in one case, patch repair using the left renal vein in one case, bypass using the splenic artery for SMA repair in one case and CA repair in one case, and bypass using the saphenous vein for the SMA in one case. The need for vascular surgical assistance was mentioned in six cases.

No revascularisation was attempted in two patients. In one patient, no revascularisation was attempted since the diagnosis was made 36 hours post-operatively and multiorgan failure had already occurred. The other patient developed post-operative ileus and vomiting; he was managed conservatively since post-operative computed tomography angiography showed adequate visceral perfusion through pancreaticoduodenal collateral flow, representing an exceptional scenario of sufficient collateral reserve.

### Patient outcome

Among the 20 patients, there were three deaths (15%): a delayed diagnosis of SMA and CA injury was made in two patients (36 and 48 hours post-operatively). The diagnosis of SMA and CA injury was made intra-operatively in the other patient, but multiorgan failure occurred later. Among 13 survivors with reported follow up (range one month to six years), vascular patency was documented as satisfactory on serial imaging.

### Quality of the included studies

Among the 16 included studies, quality was variable, since the CARE score ranged 4 – 8 (median 7). The score of each study is presented in Table 1.

## Discussion

This systematic review confirms that SMA and CA injury during nephrectomy carries high mortality rate, especially when diagnosis is delayed. This highlights the importance of anatomic basis and recognition of risk factors, early diagnosis and prompt revascularisation.

### Anatomic basis and risk factors

The anatomic basis for SMA and CA injury during nephrectomy lies in the close proximity between these vessels and the LRA ostium. The SMA originates from the anterior wall of the aorta, immediately below the CA. The LRA originates from the anterolateral aortic wall in 52% of cases and from the lateral wall in 45%.^4^ Cadaveric and computed tomography studies report close proximity between these ostia, never exceeding 10 mm, with a mean SMA to LRA distance of approximately six mm on computed tomography.^4^^,^^22^

In normal anatomy, the LRA lies posterior to the left renal vein. The CA and SMA, which arise from the anterior aortic wall, are normally separated from the hilar dissection field. However, large tumours or bulky lymphadenopathy may distort this anatomy, bringing the SMA and even the CA into the operative field where they can be mistaken for the LRA. Catarci *et al.* describe this pathogenic mechanism as a perceptive deficit similar to the Kanizsa optical illusion: the surgeon's visual system constructs a complete anatomic image from partial information, making what is perceived as the LRA look and behave convincingly like the target vessel.^2^ Accordingly, during left nephrectomy, the surgeon determines a lesion of the SMA and the coeliac trunk (CT) convinces them that they are instead dealing with LRA or its branches. This mechanism is heightened by large tumour size (> 10 cm), which mechanically distorts hilar anatomy, and by bulky para-aortic lymphadenopathy enveloping and displacing the SMA. The case reported by Anwar *et al.*, involving a bulky mass exceeding 11 cm with massive peri-aortic adenopathy, exemplifies this mechanism at its most extreme.^21^

### Critical importance of early recognition

The timing of diagnosis is crucial. Intra-operative change in bowel colour or progressive haemodynamic instability after renal hilar dissection should prompt quick vascular surgeon assistance. Post-operatively, any unexplained abdominal distension, metabolic acidosis, or progressive haemodynamic instability should also raise the suspicion of CA and or SMA injury and that a vascular surgeon may be required.

### Revascularisation techniques and surgical decision making

Revascularisation techniques will vary depending on the anatomic location of the injury. Zone 1 SMA lesions (between the aortic origin of the SMA and the emergence of the inferior pancreaticoduodenal artery) are the most common and most severe. The collateral circulation with the CT through the upper pancreaticoduodenal arches and the lower pancreaticoduodenal arches is compromised, leading to residual perfusion of the SMA territory only through the existing collateral circuits with the inferior mesenteric artery. Concomitant damage to the SMA and CT is less frequent, but results in an increased intestinal ischaemia risk. When the lesion is diagnosed early intra-operatively, it is recommended to involve a vascular surgeon for prompt revascularisation.^2^

Primary end to end anastomosis of the damaged segment is the most common revascularisation technique performed. However, this technique requires an arterial stump of sufficient length, as well as a patent and healthy downstream artery. When sufficient length is not available, the remaining LRA stump can be used, or different substitutes, such as internal iliac artery (there is however a risk of colonic ischaemia) or saphenous vein (when available and suitable).^6^^,^^9^^,^^11^^,^^14^ The splenic artery can also be used;^11^^,^^12^ because it has natural inflow, only a single anastomosis at the outflow vessel (SMA or CA) is necessary. This shortens the duration of the procedure and makes cross clamping of the aorta unnecessary. Most individuals will have adequate collateral supply to the spleen via the short gastric arteries, lessening the risk of splenic ischaemia and therefore the possible need for splenectomy.

One important notion is that synthetic bypasses should be avoided, since ischaemia can lead to bacterial translocation, with subsequent risk of graft infection. Moreover, if the lesion is diagnosed later in the post-operative period after intestinal ischaemia, necrosis and even peritonitis may have occurred. Combined revascularisation with resection of the ischaemic intestinal tract is then required.

### Preventive strategies

Prevention begins with pre-operative imaging. Computed tomography angiography with three dimensional reconstruction allows identification of the renal artery number and course and enables systematic assessment of CA-SMA-LRA spatial relationships.^3^^,^^4^ The increasing availability of augmented reality navigation tools offers promising future applications for real time intra-operative vessel identification.^23^

Intra-operatively, early identification of the aorta and systematic exposure of both the SMA and LRA before any clipping or ligation is the most reliable preventive strategy. When large lymph nodes are encountered at the renal hilum, the surgeon should stop and identify the SMA origin before proceeding with lymphadenectomy. The use of indocyanine green fluorescence angiography, recently proposed by Anwar *et al.*, may offer real time vessel identification in cases where the anatomy is distorted.^21^

When injury is suspected, prompt vascular surgeon assistance should be sought. A standing protocol for emergency vascular support during complex left nephrectomies, particularly those involving large tumours or bulky lymphadenopathy, should be considered a standard of care.

### Limitations

This review has several limitations. Firstly, it was based exclusively on case reports and small series, of variable quality. Furthermore, it is well recognised that surgeons are more probably to report their successes than their failures, and SMA and CA injury during nephrectomy might be under reported. Secondly, data completeness was highly variable across reports: exact ischaemia time, call for vascular surgical assistance, and follow up durations were missing in a significant proportion of cases. Finally, the heterogeneity of the patient populations, from neonates with Wilms tumour to adults with large renal cell carcinoma, limits the generalisability of pooled findings.

### Conclusion

SMA and CA injury during nephrectomy are rare, exclusively left sided, and carry a high mortality rate. They arise from a predictable combination of anatomic factors, large tumours or adenopathy distorting the operative field, and a cognitive mechanism of misperception that is shared with other major iatrogenic arterial injuries. The key determinant of survival is intra-operative recognition followed by immediate vascular repair. Immediate vascular surgical support is mandatory in order to provide prompt vascular repair, such as end to end anastomosis or bypasses using autologous grafts such as internal iliac artery, saphenous vein, or splenic artery.

## Funding

This research did not receive any specific grant from funding agencies in the public, commercial, or not for profit sectors.

## Declarations of interest

None.
